# Tissue donations for multiple sclerosis research: current state and suggestions for improvement

**DOI:** 10.1093/braincomms/fcac094

**Published:** 2022-04-19

**Authors:** Patrick Vanderdonckt, Francesca Aloisi, Giancarlo Comi, Alexander de Bruyn, Hans-Peter Hartung, Inge Huitinga, Tanja Kuhlmann, Claudia F. Lucchinetti, Imke Metz, Richard Reynolds, Hans Lassmann

**Affiliations:** 1 Department of Neurology, AZ Groeninge, Kortrijk, Belgium; 2 Department of Neuroscience, Istituto Superiore di Sanità, Rome, Italy; 3 Centro Sclerosi Multipla Ospedale Gallarate and European Charcot Foundation, San Rafaele Scientific Institute, Milano, Italy; 4 Department of Neurology, UZ Leuven, Leuven, Belgium; 5 Department of Neurology UKD, Germany Medical Faculty, Heinrich Heine Universität, Düsseldorf, Germany; 6 Brain and Mind Center, University of Sydney, Camperdown, Australia; 7 Department of Neurology, University of Vienna, Wien, Austria; 8 Department of Neuroimmunology, Netherlands Institute for Neuroscience, Amsterdam, The Netherlands; 9 Institut für Neuropathologie, Universitätsklinikum Münster/UKM, Münster, Germany; 10 Department of Neurology, Mayo Clinic, Rochester, MN, USA; 11 Institute of Neuropathology, University Medical Center, Göttingen, Germany; 12 Department of Brain Sciences, Imperial College, London, UK; 13 Center for Brain Research, Medical University of Vienna, Wien, Austria

**Keywords:** multiple sclerosis, tissue donation, biopsies, autopsies, brain banking

## Abstract

Although major progress in multiple sclerosis research has been made during the last decades, key questions related to the cause and the mechanisms of brain and spinal cord pathology remain unresolved. These cover a broad range of topics, including disease aetiology, antigenic triggers of the immune response inside and/or outside the CNS and mechanisms of inflammation, demyelination neurodegeneration and tissue repair. Most of these questions can be addressed with novel molecular technologies in the injured CNS. Access to brain and spinal cord tissue from multiple sclerosis patients is, therefore, of critical importance. High-quality tissue is provided in part by the existing brain banks. However, material from early and highly active disease stages is limited. An initiative, realized under the patronage of the European Charcot Foundation, gathered together experts from different disciplines to analyse the current state of multiple sclerosis tissues collected post-mortem or as biopsies. Here, we present an account of what material is currently available and where it can be accessed. We also provide recommendations on how tissue donation from patients in early disease stages could be potentially increased and for procedures of tissue sampling and preservation. We also suggest to create a registry of the available tissues that, depending on the source (autopsy versus biopsy), could be made accessible to clinicians and researchers.

## Teaser

Vanderdonckt *et al.* describe the characteristics and potential value of autopsy brain tissues collected in brain banks and neuropathological archives and brain biopsies for multiple sclerosis research. The authors provide suggestions on how to increase the availability of suitable material which may lead to new insights into disease causes and mechanisms.

## Graphical Abstract

**Graphical Abstract:**
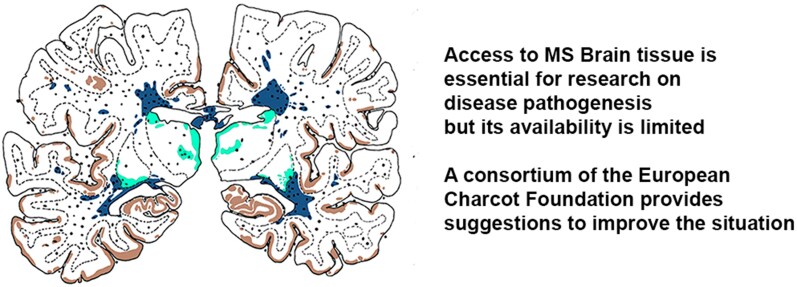

## Introduction

Translational research on the aetiology and pathogenesis of diseases of the CNS is to a large extent performed in experimental models. This is also the case in the study of multiple sclerosis. Experimental models of autoimmune encephalomyelitis, virus-induced inflammatory demyelination and toxic myelinopathies, cover the spectrum of disease mechanisms only to a limited degree. Thus, it is mandatory to test new hypotheses directly in human pathological tissues.^1^ So far, this has been carried out by applying molecular and immunological techniques on suitable material, which is available in neuropathological archives and brain banks.^2^ However, cases with highly inflammatory active lesions representative of early disease stages are rare in brain bank collections and, when present in neuropathological archives, are mainly available as formaldehyde-fixed paraffin-embedded (FFPE) tissue. In contrast, state-of-the-art molecular technologies, such as single-cell RNA sequencing, spatial transcriptomics, proteomics and metabolomics and some immunological techniques currently require well characterized and optimally preserved fresh-frozen native material (Table 1).^2–7^

**Table 1:** Central nervous system (brain and spinal cord) tissue processing: compatibility with different techniques used in multiple sclerosis research

Formaldehyde-fixed/paraffin-embedded	Fixed frozen	Snap frozen
Ideal for: Neuropathological assessment Histological stains RNA detection using in situ hybridization, RNA scope Many immunohistochemical procedures and some immunofluorescence stains Histo-cytometry Multiplex immunofluorescence imaging Potential for: Mass cytometry (CyTOF) and imaging mass cytometry (IMC) Not optimal for: Gene expression studies using real time RT-PCR, unbiased transcriptome approaches on bulk tissue, scRNA-seq, snRNA-seq, proteomic analysis	Ideal for: RNA detection using in situ hybridization, RNA scope Most immunohistochemical/immunofluorescence stains Histo-cytometry Multiplex immunofluorescence imaging Good for: Neuropathological assessment Histological stains Potential for: Mass cytometry (CyTOF) and imaging mass cytometry (IMC)	Ideal for: Analysis of single and multiple target genes in laser capture microdissected bulk tissue using real time RT-PCR Unbiased transcriptome analysis of microdissected bulk tissue scRNA-seq; snRNA-seq Spatial transcriptomics *In situ* pentamer binding Isolation of CNS-infiltrating immune cells for flow cytometry; scRNA-seq; snRNA-seq and *ex vivo* functional studies Unbiased proteomic, mass spectrometry and multiplex protein analysis Suitable but not ideal for: Immunohistochemical/immunofluorescence stains (detection of most molecules requires tissue post-fixation) *In situ* hybridization and RNA scope Not optimal for: Neuropathological assessment Histological stains

Although the collection of formaldehyde-fixed and embedded tissue is easy and feasible under all circumstances, its suitability for modern molecular technologies is currently limited. Substantial progress has been made during the last years to improve the use of these technologies in archival material.

CyTOF, cytometry by time of flight; scRNA-seq, single-cell RNA sequencing; snRNA-seq, single nucleus RNA sequencing; scTCR-seq, single-cell T-cell receptor sequencing; RT-PCR, reverse transcription-polymerase chain reaction.

To improve the provisions of post-mortem tissue, a team of clinical and basic scientists has been gathered under the patronage of the European Charcot Foundation (see Appendix I) to improve access to suitable brain tissue, in particular from multiple sclerosis patients at early disease stages and with severe disease courses. The aim of this initiative is to provide information on what material is currently available, where it is located, how it can be distributed to optimize collaborative research projects, and how it matches the demand in relation to the current technical progress in molecular biology, immunology and genomics. Based on the analysis of the current state, suggestions are provided regarding how the gap between tissue demand and availability could at least in part be bridged. The information provided here comes from the specific expertise of the panel members in multiple sclerosis research, neuropathology and brain banking and through the search of the relevant articles in PubMed.

## Gaps in knowledge related to multiple sclerosis pathology and disease mechanisms

Multiple sclerosis is an inflammatory demyelinating disease of the CNS. The pathology gradually changes with the age of the patients and disease duration.^8,9^ Early disease stages are dominated by focal lesions mainly located in the white matter, which are associated with sequential waves of inflammation, characterized by migration of circulating leucocytes (mainly lymphocytes) into the brain and spinal cord. This is accompanied by profound blood–brain barrier damage, as reflected by contrast enhancement on MRI. Primary demyelination is the cardinal pathological alteration, while axons are partially preserved. Even in lesions, where myelin is digested within the infiltrating macrophages, remyelination can be extensive.^10,11^ This type of pathology is reflected clinically by relapses and remissions of the disease. As new lesions form, the blood-derived cellular elements of the inflammatory response become trapped, mainly around the Virchow Robin spaces of large periventricular veins^12^ and in the meninges.^13,14^ The inflammatory reaction is associated with a slow expansion of a subset of pre-existing lesions in the white matter and cortex in concert with diffuse neurodegeneration in the normal-appearing white and grey matter.^15–17^ This type of pathology is evident already in the relapsing stage of the disease and gradually accrues with disease evolution. The slow accumulation of neural cells and axonal loss manifests clinically as a progressive disease when the threshold of functional compensation is passed.^9^

Although these pathologic changes in the brain and spinal cord of multiple sclerosis patients are well understood, fundamental questions regarding their initiation are unresolved. What triggers the inflammatory reaction? What antigen is recognized by T- and B-lymphocytes, propagates the immune response and sustains inflammation leading to demyelination and neurodegeneration? Is there any mechanistic role for infectious agents in the disease process, for instance, the Epstein–Barr virus, which has been causally linked to multiple sclerosis through epidemiological studies?^18,19^

Such questions can be directly addressed in patient-derived tissues in a hypothesis-driven manner or using global unbiased discovery strategies relying on state-of-the-art multi-omics developments. These research efforts are only possible when well characterized human tissue (biopsy and autopsy) collected during active disease stages is available, processed and preserved in a suitable manner. These tissue samples not only have to cover the entire spectrum of multiple sclerosis but also need to include optimally characterized and similarly processed samples from other inflammatory and neurodegenerative diseases and healthy age- and sex-matched control cases.^20–22^

## State of the current collection of brain tissue from multiple sclerosis patients

Human brain tissue becomes available through two different sources: post-mortem tissue, which is collected after the patient's death and stored in the archives of specialized brain banks or pathology departments; and biopsy material that is obtained strictly for diagnostic reasons when an alternative diagnosis is suspected.

### Autopsy brain tissue contained in brain banks

Autopsy tissue mainly, but not exclusively, becomes available from patients with long-standing disease while tissue samples from initial relapsing–remitting disease stages are rare.^17,23,24^ Depending on the analysed cohort, a variable proportion of lesions are active or mixed active/inactive, as defined by the presence of activated macrophages/microglia, but can be also nascent active demyelinating with early myelin degradation products found within macrophages.^23–26^ More rarely, patients may have a short, aggressive disease course with active clinical progression and preponderance of active lesions at the time of death.^23^ Brain tissues from these very severe cases are mainly contained in historic collections since the death in the course of acute multiple sclerosis has become very rare over the last decade as the result of effective anti-inflammatory treatments and intensive care support. Besides, acute multiple sclerosis cases can pathologically mimic some features of gliomas, misdiagnosis being a common cause for medical-legal litigation. As such, this type of material is not readily available for research.

Several multiple sclerosis brain banks have been established internationally during the last decades, mainly through the initiatives of National Multiple Sclerosis Societies (Table 2). The two largest brain banks are the Netherlands Brain Bank (www.brainbank.nl) and the UK Multiple Sclerosis Society Brain Bank (www.imperial.ac.uk/medicine/multiple-sclerosis-and-parkinsons-tissue-bank). Through well-defined donor recruitment schemes, predefined autopsy procedures and detailed protocols for tissue collection, preservation and distribution, these resources provide a wide spectrum of diverse lesion types and stages from a broad spectrum of cases together with extensive clinical information. Owing to the genuine interest of the brain bank teams in multiple sclerosis research, detailed qualitative and quantitative information on the status of inflammation, the composition of the immune cell infiltrates, demyelination and neurodegeneration are available.^17,24,27–29^ A major asset of brain banks with prospective tissue sampling is that the material can be collected for a very broad range of different research applications allowing flexibility in the use of different techniques. Thus, tissue is preserved for techniques that require fresh-frozen, paraformaldehyde-fixed and frozen or FFPE material. Furthermore, not only brain tissue but also peripheral nerves, cerebrospinal fluid and lymphoid tissues can be retrieved, and tissue collection can be expanded upon request. For specific research questions, freshly isolated inflammatory blood-derived or CNS resident cells are also available. Thus, one could argue that many of the needs for cutting-edge multiple sclerosis research are already met by the existing brain banks.

**Table 2:** Brain banks with a focus on multiple sclerosis

Brain bank	Contact
MS Society Brain Bank UK	http://www.imperial.ac.uk/medicine/multiple-sclerosis-and-parkinsons-tissue-bank/
Netherlands Brain Bank	http://www.brainbank.nl/
Rocky Mountain MS Center Tissue Bank	www.mscenter.org/research/tissue-bank
Human Brain and Spinal Fluid Resource Center, UCLA	brainbank.ucla.edu
The Harvard Brain Tissue Resource Center at McLean Hospital	www.mcleanhospital.org/research/brain-bank
NIH Neurobiobank Network	https://neurobiobank.nih.gov/
Yale University Brain Bank	https://medicine.yale.edu/lab/pitt/bank/
BrainNet Europe	http://www.brainnet-europe.org
MS Brain Bank Australia	https://msbrainbank.org.au/request-tissue/
German MS Brain Bank	https://www.kompetenznetz-multiplesklerose.de/patienteninformationen/aktuelle-studien/ms-brain-bank/; https://neuropathologie.umg.eu/forschung/forschungsschwerpunkte/ms-brainbank/

List of current brain banks, specialized in the collection of multiple sclerosis tissue samples with the respective contact information. Amount and type of the material collected in the respective brain banks can be seen in their homepages. The two largest banks internationally are the UK MS Society Brain Bank and the Netherlands Brain Bank. They also provide the broadest spectrum of tissue samples from different disease stages and with different modes of tissue preservation. Other brain banks, such as the Brain Net Europe are virtual brains banks, providing information on the collected tissues in the archives of neuropathology units.

However, there are limitations to banked tissues. The first is related to the prospective sampling of tissue from patients who have signed up for donation. As a result of this process, a large proportion of donors are patients with advanced chronic disease, where early pathological mechanisms cannot be addressed. The second problem relates to the fact that exact phenotyping and staging of multiple sclerosis lesions is mandatory for most studies of multiple sclerosis pathogenesis, but this is time-consuming and more difficult in fresh-frozen tissue samples. Thus, results obtained with new molecular or immunological technologies requiring fresh tissue may be accompanied by some uncertainty, limiting correlation to exact histopathological features. To date, spatial transcriptomics and proteomics have not overcome this hurdle. One way to define the underlying pathology would be to analyse mirror blocks of fixed and fresh-frozen tissue, respectively.^2^ Such assessment is not provided on a regular basis by the brain bank teams. The majority of studies now require frozen tissues and the respective funds for financial compensation to incorporate new and additional ways of processing would have to be provided through research projects by the groups who request such material.

A major additional problem of tissue collection in brain banks is the lack of appropriately processed tissues from a broad spectrum of control cases. Healthy age- and sex-matched controls are included by recruitment of patient's family members and some disease controls can be obtained through brain banks focusing on other diseases.^30^ However, other chronic inflammatory diseases, which are particularly relevant for comparison with multiple sclerosis, are typically not contained in the available frozen tissue collections and can only be obtained as rare archival cases.

### Autopsy tissue collected in diagnostic neuropathology units

Well-suited material for research also becomes available through autopsies performed for diagnostic reasons. The implementation of the autopsy and the use of such material for research are subject to different national regulations and local circumstances. Such autopsies are either performed when doubts exist regarding diagnosis or therapy, or when there is a specific agreement to a research donation. Collected and stored in the archives of the pathology departments for decades, a very broad spectrum of material has accumulated, covering not only the entire disease spectrum of multiple sclerosis but also the broader spectrum of vascular, inflammatory and neurodegenerative diseases for comparison. As an example, this approach has facilitated studies showing that band-like demyelination in the cerebral cortex is specific for multiple sclerosis, and not present in any other human disorder of the CNS.^22^ The neuropathology units that have collected the material can be identified through the respective publications. Major efforts are currently underway to adapt new molecular and immunological techniques for use in FFPE tissues. For example, recently it was possible to resurrect a pathogenic autoantibody response against myelin oligodendrocyte glycoprotein from the paraffin-embedded brain tissue of a patient who died more than 50 years ago.^31^ Similarly, a comparison of global gene expression in archival brain tissue from cases with multiple sclerosis and other chronic CNS inflammatory diseases has provided evidence for oxidative injury as an important driving force of demyelination and neurodegeneration in multiple sclerosis lesions.^21^

As a major limitation, such archival material is generally restricted to FFPE brain and spinal cord tissue. It is in fact unusual to have an infrastructure in place equipped for the broad spectrum of tissue preservation that is achieved in brain banks. Using highly sensitive and specific immunohistochemical and *in situ* hybridization techniques, valuable information can be gained from such material.^32^ Additional problems encountered by molecular studies using human autopsy material are related to the preservation of the tissue. Pre-mortem hypoxic brain damage is deleterious for any post-mortem molecular investigations. In addition, a major delay in collecting samples post-mortem affects the quality of the tissue, and rapid autopsy procedures are costly. Since other pathologies impact on the studies of the multiple sclerosis brains, neuropathological analyses of putative comorbid pathologies, such as vascular lesions or age-related neurodegeneration, need to be performed. Clinical information is crucial for the interpretation of the pathological data and retrospective collection and processing of clinical data is labour-intensive and costly.

### Brain biopsies

Brain biopsies with a neuropathological diagnosis of multiple sclerosis-like inflammatory demyelinating disease are rare. Biopsies are taken for diagnostic reasons and the most prevalent indication is the presence of a large tumefactive lesion in the white matter which, with an estimated incidence of 0.3/100 000 cases/year, may turn out to be due to inflammatory demyelinating disease.^33–36^ The pathology of such cases shows variable inflammation, active demyelination (characterized by the presence of macrophages/microglia containing myelin degradation products), partial axonal preservation and reactive gliosis. Although axons are spared in comparison to the complete loss of myelin, prominent acute axonal injury is common. Thus, the vast majority of these lesions resemble those seen in autopsies of acute or subacute multiple sclerosis.

Diagnostic brain biopsy may be performed in patients presenting with an acute leukoencephalopathy of uncertain aetiology. The biopsy is mainly carried out in patients with progressive tumefactive white matter lesions, which may occur in a variety of different immune-mediated, demyelinating, infectious or neoplastic conditions (Table 3). Brain biopsy will only rarely be considered in large specialized units, where the entire diagnostic armamentarium, as listed in Tables 4 and 5, is available and applied. However, such a diagnostic procedure may not always be possible in small institutions, and time constraints may dictate the early decision to resort to biopsy diagnostics. Importantly, we recommend that in all cases a basic set of diagnostic procedures should be performed, including a careful clinical examination, MRI, analysis of the CSF including markers for intrathecal immunoglobulin production, determination of serum autoantibody titres (myelin oligodendrocyte glycoprotein and aquaporin 4) and microbiological and virological analyses.

**Table 3:** Differential diagnosis in patients with tumefactive lesions in the brain or spinal cord

Type of disease	Disease	IDM in pathology
Neoplasms	Glioma	No
	Metastasis	No
	CNS lymphoma	Sometimes (Sentinel Lesions)
Infectious	Cerebritis, cerebral abscess	No
	HIV encephalitis	No
	PML	Yes
	Hepatitis C	?
Inflammatory	Sjögren's syndrome	No
	Systemic lupus erythematosus	No
	Neuro-Behçet	No
	Vasculitis: primary or secondary	No
	TNF-receptor blockade	Yes
	Graft versus host disease	Sometimes
Inflammatory demyelinating diseases	Tumefactive MS	Yes
	Baló's concentric sclerosis	Yes
	NMOSD	Yes, but initial astrocytopathy
	MOGAD	Yes
	ADEM	Yes
Genetic/metabolic	Adrenoleukodystrophy	Yes
	Alexander's disease	No

There are several different diseases which may give rise to tumefactive lesions within the central nervous system. Thus, careful clinical evaluation, magnetic resonance imaging and the use of paraclinical markers in serum and cerebrospinal fluid is important, before a CNS biopsy is considered. In brain biopsies, multiple sclerosis may present with a pattern of inflammatory demyelination. Therefore, careful neuropathological analysis in the context of the clinical presentation has to be performed to reach a final diagnosis. In addition, careful clinical follow-up is necessary. If this is not done, research on such material may lead to conclusions which are not relevant for multiple sclerosis.

PML, progressive multifocal encephalopathy; NMOSD, neuromyelitis optica spectrum disorders; MOGAD, myelin oligodendrocyte glycoprotein antibody-associated disease; ADEM, acute disseminated encephalomyelitis.

**Table 4:** Clinical and laboratory analysis of patients with multiple sclerosis, including those with tumefactive lesions

*Clinical data*
Demographic data Medical history: Prior multiple sclerosis diagnosis? Comorbidities? Immunocompetent state? Vaccination status? Recent vaccinations? Medication use Familial history Symptoms and clinical examination: Age at presentation Presenting neurologic symptoms System anamnesis including environmental and professional exposure Current EDSS + functional system scores Current GCS Current Modified Ranking scale Previous treatments: Immune modulating treatment? Corticosteroids PLEX?
*Evoked potentials*
VEP: prolonged P100 latency (present in ∼1/3 cases of pathology proven demyelinating origin) SSEP: prolonged/absent cortical response (present in ∼60% of cases of pathologically proven demyelinating origin)
*Laboratory testing*
Basic haematology, kidney function, ionogram, liver function, C-reactive protein, TSH ANA, ANCA, RF, erythrocyte sedimentation rate, complement, lupus anticoagulants, anti-cardiolipin antibodies, serum electrophoresis, ACE, sedimentation, anti-AQP4 antibodies, anti-MOG antibodies, HIV, HBV, HCV, toxoplasma serology, CMV serology, VZV serology, EBV serology, syphilis serology, Borrelia serology, tumour markers, JCV titre, IGRA test Neurofilament light protein
*Lumbar puncture*
Lumbar puncture performed? If yes, date, previous therapies? If no, reason? Cell number, protein, glucose, lactate Flow cytometric immunophenotyping for haematological malignancies IgG index; oligoclonal bands (OCB) JC virus PCR
*References*: Lucchinetti *et al.*^33^; Kuen *et al.*^37^; Wattamwar *et al.*^38^; Algahtani *et al.*^35,36^

This table summarizes the information which should be available from patients with multiple sclerosis, including those with tumefactive lesions, who donated CNS tissue for research. This applies for autopsy and biopsy tissue.

EDSS, expanded disability status score; GCS, global composite score; PLEX, plasma exchange; VEP, visual evoked potentials; SSEP, somatosensory evoked potentials; ANA, anti-nuclear antibodies; ANCA, anti-neutrophils cellular antibodies; RF, rheumatoid factor; ACE, angiotensin-converting enzyme; AQP4, aquaporin 4; HBV, hepatitis B virus; HCV, hepatitis C virus; VZV, varicella zoster virus; EBV, Epstein–Barr virus; JCV, John Cummings virus; IGRA test, interferon gamma release assay test (tuberculosis); OCB, oligoclonal bands.

**Table 5:** Neuroimaging in patients with tumefactive multiple sclerosis CNS lesions

*Brain imaging*
MR
Location of lesions: Frontal, parietal, temporal, occipital, deep grey matter, cortical, infratentorial? Number of lesions Mass effect (45–71% cases) Perilesional oedema (77–100% cases) Gd enhancement (75–95% cases) Closed ring Open ring (sens 71.4%, spec 98%) Heterogeneous enhancement (patchy, nodular, punctate) Perfusion imaging performed mean relative cerebral blood volume within tumefactive demyelinating lesions have been found to be substantially less than in high-grade gliomas and lymphomas Corpus callosum involvement Presence of T_2_-weighted hypointense rim co-localizing with ring enhancement (33–79% cases) Presence of peripheral restricted diffusion on DWI Presence of other non-tumefactive typical multiple sclerosis lesions (50–65.5% cases): Periventricular, juxtacortical, infratentorial, cortical? Presence of central vein sign? yes/no Iron ring lesions
Magnetic spectroscopy
Increased Cho/Cr ratio: Cho/NAA ratio: cut-off of Cho/ NAA ratio of >1.72 is an indicator of high-grade gliomas rather than tumefactive demyelinating lesions^39^ Reduced NAA/Cr ratio: yes/no Increased glutamine and/or glutamate peak: yes/no
*Brain PET imaging*
FDG-PET: Increased metabolism (relatively less versus glioma's) Persistent hypermetabolism after treatment with corticosteroids favours diagnosis of primary central nervous system glioma or lymphoma C-Methionine PET: yields higher sensitivity (93%) and specificity (78%) to differentiate high-grade gliomas from non-neoplastic lesions, including TDLs, when *T*/*N* ratio is over 2.0 Persistent hypermetabolism after treatment with corticosteroids favours diagnosis of primary central nervous system glioma or lymphoma
*Spinal cord imaging*
Date of MR scan Presence of short focal T2 lesions: yes/no Presence of LETM: yes/no Presence of Gd enhancement?
*Other*
CT thorax/abdomen FDG-PET full body Non-CNS biopsy results
References: Lucchinetti *et al.*^33^; Kiriyama *et al.*^40^; Totaro *et al.*^34^; Algathani *et al.*^35,36^; Ikeguchi *et al.*^39^

This table summarizes the information which should be available from patients who donated CNS tissue for multiple sclerosis research. This applies for autopsy and biopsy tissue.

DWI, diffusion-weighted imaging; Cho, choline; NAA, n-acetyl aspartate; Cr, creatine; PET, positron emission tomography; TDL, tumefactive demyelinated lesion; LETM, longitudinally extensive transverse myelitis; Gd, gadolinium.

When a biopsy is considered, the procedure should be stringently coordinated between the neurologist, the neuroradiologist, the neurosurgeon and the neuropathologists. In general, only small stereotactic needle biopsies are taken, since they are in most instances sufficient to reach a final diagnosis. Care must be exercised to ensure that samples of the perilesional tissue, the lesion edge and the lesion centre are available. It is not sufficient to select the areas just on the basis of T_2_-weighted MRI scans, since MRI sequences that more reliably differentiate tissue damage from oedema are instrumental.^41^ If possible, areas with contrast enhancement should be chosen, and when clinically possible, steroids should not be administered before the procedure. The presence of active lesions provides information on the mechanisms of demyelination (e.g. inflammatory versus non-inflammatory), which may not be apparent in inactive plaques. Steroid treatment prior to biopsy changes the composition of inflammatory infiltrates and may obscure the differential diagnosis with lymphoma.^42^ Currently, most of the tissue obtained during brain biopsy is immediately fixed with formaldehyde in the operation theatre and then embedded in paraffin. However, molecular analysis of the tissue has gradually gained acceptance in the field of oncology^43^ and this is also the case for infectious diseases. Thus, it is preferable that future biopsy material will be partly fixed and embedded for routine neuropathological diagnostics and other pieces will be flash-frozen for biochemical and molecular analysis. Obviously, this approach is limited by the primary effort to restrict the size of the removed tissue as much as possible. Open biopsies are indicated in some scenarios when meninges need to be sampled, after non-diagnostic stereotactic biopsy, or when surgical resection of a presumed neoplasm is carried out.

The aim of neuropathological analysis is to establish an accurate diagnosis. A practical guide to reach this goal in the analysis of tumefactive demyelinating lesions is provided by Kuhlmann *et al.*^42^ When the diagnosis is established, research can be performed on the biopsy tissue on the following provisions:

1. Suitable material is stored only after finalizing the diagnostic procedure. However, it must be considered that some of the leftover material has to be held back for future diagnostic work or for legal reasons. As the initial biopsies are typically only few millimetres in size, typically no material can be spared.
2. The use of biopsy material for research is subject to national ethical and legal regulations. An ethics committee approval and informed consent from the patient are required.

Brain and spinal cord biopsies are very attractive for multiple sclerosis research because they are mainly performed at very early disease stages and may provide insights into the initial immunopathologic processes. In addition, when properly handled, biopsies are not hampered by pre- and post-mortem conditions, which frequently prohibit more sophisticated molecular analysis. Another advantage is that extensive clinical and paraclinical data can be gathered, blood samples can be taken and clinical follow-up investigations performed, allowing to conduct valuable pathological–clinical–serological–radiological correlations.^33,44,45^

However, the use of biopsy tissue for multiple sclerosis research is limited by several factors:

1. Research making use of biopsy tissues mainly relies on specimens that are already available for diagnostic reasons.^25^ As an example, a detailed quantitative analysis of oligodendrocyte or axon loss in actively demyelinating lesions can be performed on such material.^10,46^ However, additional research is difficult due to the extremely limited amount of tissue available.
2. So far, the vast majority of biopsies are available as an FFPE material, since this is the basic methodology for diagnostic neuropathology. Methods of spatial transcriptomics or proteomics may become important diagnostic tools in the future when it is shown that their diagnostic accuracy is similar to or even better compared with the well-established conventional techniques. An alternative approach is to optimize the protocols enabling the use of new technologies in archival paraffin material. This is in principle possible, but major technical improvements are necessary to obtain results that are comparable with those gathered in unfixed native material.^47^
3. Multiple sclerosis lesions have a very complex 3D architecture and a meaningful interpretation of molecular changes in a lesion requires that they are evaluated in the proper spatial and temporal context of lesion evolution.^48^ This information is best obtained from large hemispheric and serial brain sections than from single sections of a given lesion and minuscule biopsy tissues.
4. Pathology can classify a tumefactive lesion in the white matter as inflammatory demyelinating, but this alone does not provide final proof that the patient has multiple sclerosis. There are several other conditions that can lead to inflammatory demyelination. These conditions include neuromyelitis optica spectrum disorder,^49^ myelin oligodendrocyte glycoprotein antibody-associated disease (MOGAD),^50^ inflammatory demyelinating lesions in patients with graft versus host disease,^51^ patients receiving treatment with tumor necrosis factor (TNF) blocking agents,^52^ and patients with intracerebral lymphoma associated with sentinel demyelinating lesions.^53^ Most of these cases develop a monophasic or relapsing course without transformation into chronic progressive disease. Careful diagnostic evaluation including clinical and serological information will exclude most of these conditions, and several studies showed that the majority of patients with tumefactive inflammatory demyelinating diseases develop prototypic multiple sclerosis, defined by the McDonald diagnostic criteria.^33^ Long-term follow-up studies indicate that many of these patients will ultimately develop progressive multiple sclerosis.^54^ However, it is important to closely monitor further disease evolution in presumed multiple sclerosis patients after brain biopsy.^44,45^

## Conclusions

Existing brain banks are an excellent source of human material for research. They provide a broad range of high-quality tissue supplemented by samples of serum or plasma and CSF from patients, who have been prospectively recruited into a specific donor programme. The major limitation resides in the prospective donor-based tissue sampling, which in a disease such as multiple sclerosis results in low numbers of cases at early disease stages with rapid disease evolution. Such cases, however, are particularly attractive for research focusing on multiple sclerosis aetiology and pathogenesis.

Cases with the aggressive and rapidly evolving diseases are rare nowadays, due to improved treatment and supportive care of the patients. They are still occasionally encountered in brain banks but more often in diagnostic neuropathological units, and permission for autopsy is sometimes achieved from the patients or their relatives. Such material is in general processed to generate FFPE specimens, which allows state-of-the-art neuropathological evaluation and long-term storage. Obtaining fresh-frozen tissue material under these conditions is difficult and in general not routinely established in the involved units.

Biopsy tissue from multiple sclerosis patients is available for research rarely since in most instances very small stereotactic needle biopsies are performed. This material is suitable mainly for research questions that can be addressed by analysing sections, which are necessary and have been assessed for diagnostic neuropathology. However, even when present, leftover material has to be held back in many institutions for legal reasons. Thus, the chance that suitable additional material is accessible for research use is usually very low.

## Recommendations

### Increase the availability of brain tissue from patients with early multiple sclerosis and an aggressive disease course or cause of death unrelated to multiple sclerosis

There is a much higher likelihood to achieve this through autopsy programmes than to rely on diagnostic biopsies. Most importantly, neurologists and physicians, as well as patients and their relatives should be informed about the importance of this issue and motivated to provide permission for an autopsy. It must be made clear that available tissue will aid in the understanding of disease pathogenesis and can be used for the validation of findings in other disease models. In addition, it is of critical importance to collect autopsies from other neuroinflammatory and neurodegenerative diseases. Comparing multiple sclerosis with these diseases will provide clues on multiple sclerosis-specific disease mechanisms.

### Tissue sampling should be performed in a standardized way

As a priority, post-mortem delays should be reduced as much as possible. Furthermore, an optimized protocol of formaldehyde fixation and paraffin embedding that minimizes RNA and protein damage should be used. Ideally, tissue blocks should be preserved by snap freezing and adjacent FFPE mirror blocks should be preserved. The protocols for tissue sampling, elaborated in the existing brain banks, should be followed as much as possible. The procedures defined by the Netherlands or the UK Multiple Sclerosis Brain Banks may serve as examples (Table 6) (https://www.brainbank.nl/nbb-ms/, https://www.imperial.ac.uk/medicine/multiple-sclerosis-and-parkinsons-tissue-bank/research/). However, flexibility is necessary to adapt these protocols to meet future demands, when new technologies become available.

**Table 6:** Procedures of multiple sclerosis tissue sampling, as defined in the guidelines of major multiple sclerosis tissue banks

Autopsy procedures: Post-mortem time as short as possible (between 6 and 24 h, when possible). Determine tissue and/or CSF pH as a marker of agonal status before death as indicator of pre-/post-mortem tissue damage. Samples to be collected: Brain including optic nerves, spinal cord, post-mortem cerebrospinal fluid, plasma and eventually other tissues, such as for instance (cervical) lymph nodes or gut. Maintain intact meninges where possible. Collect demographic data, information on disease type and course and information about paraclinical investigations (with preservation of original MRI documentation and serum or CSF samples, when possible) and investigate the brain tissue for comorbid pathologies such as Alzheimer's disease and Parkinson's disease Braak stages. Take digital images of all tissues from arrival to storage. Tissue dissection and preservation: Brain dissection in coronal slices of 1 cm. Global tissue samples by immersion of some slices in 4% buffered paraformaldehyde (pH: 7.4) or 4% formalin for paraffin embedding and mirror samples as snap frozen tissue in isopentane/dry ice or liquid nitrogen. Specific sampling of multiple sclerosis lesions and normal-appearing white and cortical grey matter guided by macroscopic inspection or previous (post-mortem) magnetic resonance imaging. Sampling for cortical demyelination should take into account that cortical lesions are larger and more numerous in the infoldings of the brain surface (cortical sulci, insular cortex, limbic cortex). Separate standardly dissected blocks from the brain stem and cervical, thoracic and lumbar spinal cord and optic nerves, when available. If facilities are available, take meningeal tissue samples and culture for production of fibroblast cell lines that can be used to generate iPSC cells. Tissue that remains after dissection of all blocks should be kept in formaldehyde. Tissue preservation: Formaldehyde-fixed tissue (formaldehyde fixation time should not exceed 4 weeks): Whole-brain hemispheres embedded in paraffin. Multiple small tissue blocks of lesions and normal-appearing tissue in the white and grey matter embedded in paraffin. Paraformaldehype fixed tissue blocks, which are snap frozen after cryoprotection (30% sucrose in PBS). Dissect small snap frozen tissue blocks from hemispheric slices according to the lesions characterization and staging in the formaldehyde-fixed mirror blocks. Aliquot CSF into small samples (e.g. 200 ml) and freeze at −80°C. Isolate DNA from a small piece of frozen tissue and aliquot and freeze at −80°C. Lesion characterization Tissue embedded in paraffin form mirror blocks can be cut to double stain with HLA/PLP for lesion characterization according to Kuhlmann *et al.*^42^ scanned and these images can be used for tissue dissemination of both paraffin blocks and frozen mirror blocks.

iPSC, inducible pluripotent stem cells; MHC, major histocompatibility complex; PBS, phosphate-buffered saline; PLP, proteolipid protein.

### International documentation of sample availability

The number of tissues from early multiple sclerosis cases will never be very high since such conditions are rare. The material could either be collected in the archives of the original neuropathological departments or transferred into existing brain banks that can perform proper pathological classification of the multiple sclerosis lesions and other tissue samples, summarize retrospectively the collected clinical information, store the tissues and take care of tissue and data dissemination. However, research on single cases and/or lesions is of limited value in a disease such as multiple sclerosis with its multiple and variable phenotypes. Thus, it would be important to implement an international documentation system that provides information on what material is available and who should be contacted for collaborative studies. In addition, it would be important not only to document which tissues would be available for research projects but also to have feedback from studies that have already been concluded. As the tissue is a precious resource, by feeding back the findings collected, data could be shared instead of tissues. Such a database could be implemented with the support of the European Charcot Foundation. Although a general database for biobanking is already available (BBMRI-ERIC), its contents related to multiple sclerosis are very limited.

### Identify funding opportunities and make them available to the brain banks and researchers

It is a general problem of brain banks that provision of tissue material cannot be commercialized for ethical reasons, and research grants of brain bank users do not cover the basic costs for tissue sampling, characterization, storage and tissue dissemination. The costs for work up of a single brain vary from 10 to 15 k€.^55–57^ Thus, adequate, permanent funding, which is currently provided in part by national multiple sclerosis Societies, is necessary. One could think of additional funding sources through the European Charcot Foundation, possibly involving donations from foundations and pharmaceutical industry. This appears particularly necessary if the initiative to foster global tissue donation outside established brain banks will be successful.

### Improvement of the protocols for the study of fixed tissues using cutting-edge technologies

For practical reasons, an important part of the disease-relevant tissue from multiple sclerosis patients accumulates in diagnostic units as FFPE material. When fixation time and embedding procedures are well standardized, such material can still be analysed in studies using the most advanced genomic, transcriptomic and proteomic platforms since protocols for DNA, RNA and protein extraction from fixed archival material have been developed (see also Beltran *et al.*^31^). However, major problems remain regarding the sensitivity and specificity of these methods in fixed archival material. Future efforts should aim at improving the protocols allowing the implementation of cutting-edge technologies in archival material.

### High-quality meta-data with controlled vocabulary

For the reasons mentioned previously, biopsy material will only occasionally be available for multiple sclerosis research, mainly due to the ethical constraints that limit the amount of tissue removed. However, information about such material should also be provided within the international databanks established for autopsy tissue. The exact type of information should be agreed upon and presented in a standard fashion, including controlled vocabularies and ontologies to facilitate seamless data sharing.

### Validation of findings obtained in rare multiple sclerosis brain samples

Most patients with multiple sclerosis die during the chronic progressive phase of the disease and many cases dying at early disease stages, whose brain samples accumulate as autopsies or biopsies in brain banks or neuropathological archives, show atypical clinical or pathological features. For this reason, observations made in a single case should not be generalized, but validated in additional cases, including those with regular disease courses. Particularly, in patients with brain biopsies, careful clinical, radiological and immunological follow-up studies are necessary to understand the relevance of the findings for the general multiple sclerosis patient population.

## Supplementary Material

fcac094_Supplementary_Data

## Data Availability

Data sharing is not applicable to this article as no new original data were created or analysed in this study.

## Appendix I

Members of the European Charcot Foundation Scientific Working Group on Multiple Sclerosis Tissue Research:

Francesca Aloisi, Jorge Ivan Alvarez, James L. Bernat, Giancarlo Comi, Alexander de Bruyn, Gavin Giovannoni, Hans-Peter Hartung, Inge Huitinga, Leah Kottyan, Tanja Kuhlmann, Hans Lassmann, Claudia Lucchinetti, Roberta Magliozzi, Imke Metz, Richard Nicholas, Jean Costa Nunes, Richard Reynolds, Barbara Serafini, Anne Sieben, Patrick Vanderdonckt, Jelle Vandersteene, Matthew Weirauch and Lawrence Young.

## Abbreviations

FFPE =: formaldehyde-fixed paraffin-embedded (FFPE)
MOGAD =: myelin oligodendrocyte glycoprotein antibody-associated disease
