# Supplementary material for: Tissue donations for multiple sclerosis research: current state and suggestions for improvement
Source: Brain Commun. 2022 Apr 19;4(2):fcac094. doi: 10.1093/braincomms/fcac094 (PMC9039502; doi:10.1093/braincomms/fcac094)
Supplement: fcac094_Supplementary_Data [file fcac094_supplementary_data.docx]

| Francesca Aloisi (Department of Neuroscience, Istituto Superiore di Sanità, Rome, Italy) |
| --- |
| Jorge Ivan Alvarez (Department of Pathobiology, University of Pennsylvania, USA) |
| James L. Bernat (M.D.Professor of Neurology,Geisel School of Medicine at Dartmouth Hanover USA) |
| Giancarlo Comi (Centro Sclerosi Multipla ospedale Gallarate, European Charcot Foundation, Milano, Italy) |
| Alexander de Bruyn MD Resident Neurology-UZ Leuven, Belgium |
| Gavin Giovannoni (Blizard Institute, Queen Mary University of London, UK) |
| Hans-Peter Hartung (Deptartment of Neurology, Heinrich Heine University Düsseldorf, Germany) |
| Inge Huitinga (Department of Neuroimmunology, Netherlands Institute for Neuroscience and the Netherlands Brain Bank, Amsterdam, The Netherlands) |
| Leah Kottyan (Autoimmune Genomics & Etiology, Cincinnati Children's Hospital Medical Center, USA) |
| Tanja Kuhlmann (Universitätsklinikum Münster \| UKM · Institut für Neuropathologie, Germany) |
| Hans Lassmann (Center for Brain Research, Medical University of Vienna, Austria) |
| Claudia Lucchinetti (Department of Neurology, Mayo Clinic – Rochester, USA) |
| Roberta Magliozzi (Neurosciences - Università di Verona, Italy) |
| Imke Metz (Institute of Neuropathology, University Medical Center Göttingen, Germany) |
| Richard Nicholas (UK MS Society Tissue Bank, Imperial College, London, UK) |
| Jean Costa Nunes (Federal University of Santa Caterina, Brazil) |
| Richard Reynolds  (UK MS Society Tissue Bank, Imperial College, London, UK) |
| Barbara Serafini (Department of Neuroscience, Istituto Superiore di Sanità, Rome, Italy) |
| Anne Sieben (Department of Neurology Universital Hospital Gent / Institute Born Bunge Antwerp, Belgium) |
| Patrick Vanderdonckt (Neurology AZ Groeninge Kortrijk, Belgium) |
| Jelle Vandersteene MD (Neurosurgery University Hospital Ghent, Belgium) |
| Matthew Weirauch (Autoimmune Genomics & Etiology, Cincinnati Children's Hospital Medical Center, USA) |
| Lawrence Young (Molecular Oncology-Warwick Medical School, UK) |

**Supplementary Table 1: Members of the European Charcot Foundation Scientific Advisory Committee on MS Tissue Research**
